# Benchmarking of human Y-chromosomal haplogroup classifiers with whole-genome and whole-exome sequence data

**DOI:** 10.1016/j.csbj.2023.09.012

**Published:** 2023-09-15

**Authors:** Víctor García-Olivares, Adrián Muñoz-Barrera, Luis A. Rubio-Rodríguez, David Jáspez, Ana Díaz-de Usera, Antonio Iñigo-Campos, Krishna R. Veeramah, Santos Alonso, Mark G. Thomas, José M. Lorenzo-Salazar, Rafaela González-Montelongo, Carlos Flores

**Affiliations:** a Genomics Division, Instituto Tecnológico y de Energías Renovables (ITER), Santa Cruz de Tenerife, Spain; b Plataforma Genómica de Alto Rendimiento para el Estudio de la Biodiversidad, Instituto de Productos Naturales y Agrobiología (IPNA), Consejo Superior de Investigaciones Científicas, San Cristóbal de La Laguna, Spain; c Department of Ecology and Evolution, Stony Brook University, Stony Brook, NY 11794-5245, United States; d Department of Genetics, Physical Anthropology and Animal Physiology, University of the Basque Country UPV/EHU, Leioa, Bizkaia, Spain; e María Goyri Building, Biotechnology Center, Human Molecular Evolution Lab 2.08 UPV/EHU Science Park, 48940 Leioa, Bizkaia, Spain; f UCL Genetics Institute, University College London (UCL), Gower Street, London WC1E 6BT, United Kingdom; g Research Department of Genetics, Evolution & Environment, University College London (UCL), Darwin Building, Gower Street, London WC1E 6BT, United Kingdom; h Research Unit, Hospital Universitario Nuestra Señora de Candelaria, Santa Cruz de Tenerife, Spain; i CIBER de Enfermedades Respiratorias (CIBERES), Instituto de Salud Carlos III, Madrid, Spain; j Facultad de Ciencias de la Salud, Universidad Fernando de Pessoa Canarias, Las Palmas de Gran Canaria, Spain

**Keywords:** Next-generation sequencing, Population genetics, NRY haplogroup classification, Comparative genomics, Y chromosome

## Abstract

In anthropological, medical, and forensic studies, the nonrecombinant region of the human Y chromosome (NRY) enables accurate reconstruction of pedigree relationships and retrieval of ancestral information. Using high-throughput sequencing (HTS) data, we present a benchmarking analysis of command-line tools for NRY haplogroup classification. The evaluation was performed using paired Illumina data from whole-genome sequencing (WGS) and whole-exome sequencing (WES) experiments from 50 unrelated donors. Additionally, as a validation, we also used paired WGS/WES datasets of 54 individuals from the 1000 Genomes Project. Finally, we evaluated the tools on data from third-generation HTS obtained from a subset of donors and one reference sample. Our results show that WES, despite typically offering less genealogical resolution than WGS, is an effective method for determining the NRY haplogroup. Y-LineageTracker and Yleaf showed the highest accuracy for WGS data, classifying precisely 98% and 96% of the samples, respectively. Yleaf outperforms all benchmarked tools in the WES data, classifying approximately 90% of the samples. Yleaf, Y-LineageTracker, and pathPhynder can correctly classify most samples (88%) sequenced with third-generation HTS. As a result, Yleaf provides the best performance for applications that use WGS and WES. Overall, our study offers researchers with a guide that allows them to select the most appropriate tool to analyze the NRY region using both second- and third-generation HTS data.

## Highlights

• Whole-exome sequencing provides sufficient information to classify the NRY haplogroup.
• Among the tools evaluated, YLeaf offers the best performance.
• Nanopore sequencing technology provides enough resolution to accurately retrieve NRY haplogroup.

## Graphical abstract

**Figure:**
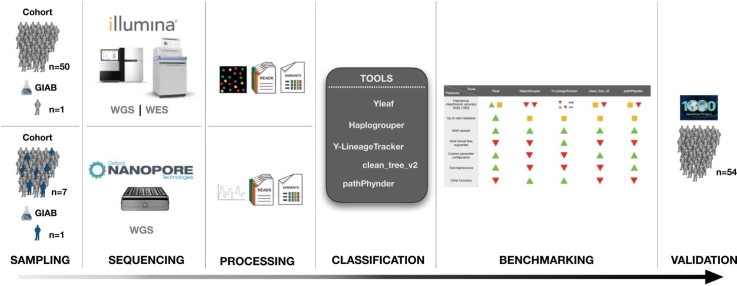

## 1 Introduction

The Y chromosome (chrY) is one of the smallest chromosomes in the human genome (∼60 Mb). A large proportion of this chromosome (95%), known as the nonrecombining region (NRY), shows patrilineal inheritance following haploid behavior due to its lack of recombination during meiosis [1]. Because of this, the NRY allows for precise reconstruction of the chrY genealogy back to a common ancestor, as described by coalescence theory [2]. Studies of the NRY have a wide range of applications in fields such as evolutionary anthropology and population history [3], [4], medical genetics [5], [6], and forensic science [7], [8].

The advent of high-throughput sequencing (HTS) technology has brought about a revolution in the development of human genomics and medicine. The decrease in costs and the increase in coverage of both whole-genome sequencing (WGS) and whole-exome sequencing (WES) applications offer the possibility of improving chrY research through deeper and better analyses [9], [10]. However, this chromosome presents regions that are challenging to sequence, such as short tandem repeats (STRs) [11], [12]. In this regard, third-generation HTS generating longer reads with greater read depths may help improve the mapping of such complex repeat sequences [10], [13]. An example of this is the use of nanopore technology (ONT, Oxford Nanopore Technologies, Oxford, UK) to successfully generate the first African human chrY reference assembly [14].

The use of HTS technology for human genome sequencing has enabled the discovery of new variants in chrY, markedly increasing the volume of marker information available to trace human paternal lineages [15]. In this regard, the International Society of Genetic Genealogy (ISOGG-Y-DNA tree; https://isogg.org/tree/) has compiled all new variants in the NRY since 2006, generating a database that currently hosts more than 90,650 unique biallelic variants. NRY diversity has been structured following a phylogenetic hierarchy based on variants that define distinct clades representing haplotypes commonly referred to as haplogroups [16]. The study of these haplogroups allows us to trace the origins and patterns of differentiation between populations and to unravel historical patterns of human migration over time [17]. Haplogroup identification is, therefore, a key step in recovering ancestral information from analyzed samples and revealing pedigree relationships whenever thorough, deep classification is feasible.

The exponential increase in the number of NRY markers, which is concomitantly associated with a rise in the complexity of the chrY tree, and the development of HTS impose a bioinformatics challenge for inferring the patrilineal genealogy of study samples. To take advantage of the potential offered by HTS technology, the number of automated NRY classification tools has seen a considerable increase in recent years [18], [19], [20], [21], [22], [23], [24], [25]. However, comparative studies evaluating the performance of each tool are lacking. Here, we present a benchmarking analysis of several command-line tools for automated human NRY classification using empirical short-read HTS data from two of the most widely used methods in human genetics, WGS and WES. In addition, we assessed the performance of the haplogroup classification tools on long, noisy, WGS read data obtained with third-generation HTS (specifically ONT).

## 2 Material and methods

### 2.1 Samples, library preparation, and sequencing

The study was approved by the Research Ethics Committee of the Hospital Universitario Nuestra Señora de Candelaria (CHUNSC_2020_95) and performed according to The Code of Ethics of the World Medical Association (Declaration of Helsinki).

Fifty DNA samples from unrelated donors were used for the study after informed consent was obtained. All samples were sequenced in parallel using short-read WGS and WES. The construction of libraries was performed with Illumina preparation kits (Table S1) following the manufacturer’s recommendations (Illumina Inc., San Diego, CA, USA). The Nextera DNA Prep Kit and Illumina DNA Prep Kit were used for WGS. The same samples were processed with the Nextera DNA Exome and Illumina DNA Prep with Enrichment Kit as described elsewhere [26]. Library quality control was carried out in a TapeStation 4200 (Agilent Technologies, Santa Clara, CA, USA), and sequencing was conducted on HiSeq 4000 or NovaSeq 6000 (Illumina, Inc., San Diego, CA, USA) instruments.

Seven of these samples were also sequenced using long, noisy WGS read data obtained with nanopore technology at KeyGene (Wageningen, The Netherlands). Sequencing was performed on a PromethION system (ONT) for 64 h using one FLO_PR002 (R9.4.1 pore) flow cell per sample following the manufacturer’s recommendations. Base calling was conducted on the PromethION computing module using MinKNOW v1.14.2 with Guppy v2.2.2, and data preprocessing metrics were calculated with PycoQC v2.5.2 [27]. Data from a reference sample from the GIAB Project (NA24385) were also included in the analysis. This sample was processed as described elsewhere and sequenced on a PromethION platform [28].

### 2.2 Bioinformatic processing

Processing of short-read WGS and WES data was carried out using an in-house pipeline based on GATK v4.1 for WGS and GATK v3.8 for WES (McKenna et al., 2010) (Fig. S1). Raw reads were assessed using FastQC v0.11.8 software [29] and aligned to the GRCh37/hg19 reference genome using BWA-MEM v0.7.15 [30]. Quality control of aligned reads was performed with Qualimap v2.2.1 [31]. The alignments were then processed for duplicate marking and base quality score recalibration [32]. The variant calling step was conducted by GATK HaplotypeCaller following the Broad Institute’s best practices workflow for germline short variant discovery. From the resulting BAM files, the NRY region (2.64–59.03 Mb) was extracted by using SAMtools v1.12 [33]. Regarding the WES data, 90% of the 676 DNA capture probes used for hybridization-based target enrichment of the chrY regions were within the NRY, covering 0.22% of this region. For ONT data, raw long, noisy reads were first preprocessed with FiltLong v0.2.1 (https://github.com/rrwick/Filtlong) to exclude reads shorter than 1000 bp (Fig. S1). The filtered reads were assessed using NanoPlot v1.38.1 [34], aligned to the GRCh37/hg19 reference genome using Minimap2 v2.22-r1101 [35] and sorted with SAMtools v1.14, extracting only reads aligned to the NRY region. The variant calling step was performed with Clair3 v0.1-r12 (https://github.com/HKU-BAL/Clair3). All these bioinformatic processes were computed using Teide-HPC infrastructure (https://teidehpc.iter.es/en/home/).

### 2.3 Sex quality control

To identify the sex of donors, quality control was performed based on both the self-reported sex of the individual and two bioinformatics approaches. The first approach, performed by Somalier v0.2.15 [36], identifies the sex of the sample from the depth of the X- and Y-chromosome reads. For the second approach, an in-house heuristic script (https://github.com/genomicsITER/sexQC-for-NGS-data) was used. The analysis involves assessing the depth of 11 genes in the nonpseudoautosomal regions of the X and Y chromosomes using high-quality mapped reads (MappingQuality>50). All samples used for this study were identified as male with consensus from these two approaches.

### 2.4 Y-chromosomal haplogroup classification

Among the tools available from the literature, we selected eight tools that were open-source and offered a command-line interface (Table 1). These were run with the 2020 version (v15.73) of the ISOGG repository database, which contains more than 90,000 polymorphic markers and constitutes the central reference used by many bioinformatic tools to classify human chrY sequences. However, three of the tools (YHap, AMY-tree, and yhaplo) were ultimately excluded from the study because they imposed limitations for database updates. For Yleaf, version 2.2 was used since the newer version 3.1 does not use ISOGG marker identifiers in the classification. Y-LineageTracker has the option of using VCF and BAM input files, fostering evaluation with the two alternative supported file types. The haplogroup classification process was executed using a workstation running CentOS 7 with 2 Intel Xeon Cascade Lake 6252 Gold CPUs at 2.1 GHz and with 384 GB of RAM. Among all the tools evaluated, clean_tree_v2, Yleaf, and pathPhynder allow the modification of certain parameters (such as base quality, depth of coverage and allele fraction) to optimize the classification process. However, since not all tools allow parameterization, we decided to run all tools using the default parameters.

**Table 1:** List of tools assessed for human Y-chromosomal haplogroup classification. All the tools assessed perform classification according to the ISOGG nomenclature by using the latest version 15.73 (2020).

**Tool**	**Release year**	**Version**	**ISOGG version (year)**	**Input options**
pathPhynder	2022	1.a	2020	BAM
Y-LineageTracker	2021	1.3.0	2019	BAM/VCF
HaploGrouper	2020	-	2019	VCF
clean_tree_v2	2019	2.0	2018	BAM
Yleaf	2018	2.2	2019	BAM
yhaplo	2016	1.1.0	2016	VCF
YHap	2013	-	2017	VCF
AMY-tree	2013	2.0	2013	VCF

Unlike WGS, which recovers a larger part of the NRY, WES only partially recovers the NRY (Table S1). This difference may lead to discrepancies in the haplogroup classification obtained by the two applications simply because it is expected that a lower level of resolution could be obtained for WES in any given sample. To address this limitation in the benchmarking, we used the maximum classification level retrieved by WES that matched the one obtained from the WGS data as the reference for comparisons.

### 2.5 Validation dataset

For validation purposes, 54 male individuals (classified as belonging to the Iberian population in Spain) from 1000 Genomes Project (1KGP) Phase 3 were evaluated to assess the performance of the different tools. WGS and WES data were obtained for all the samples in the form of BAM alignment files from the 1KGP repository [37]. The variant calling step was conducted following the previously described pipeline based on GATK HaplotypeCaller.

## 3 Results

### 3.1 Sequencing summary for short-read and long-read sequencing

**Fig. 1:**
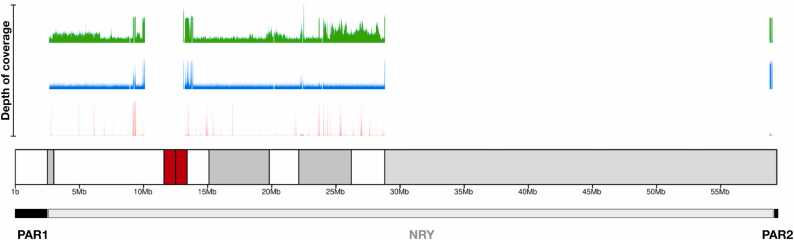
Plot of the depth of coverage for short-read and long-read sequencing in the nonrecombining portion of the Y chromosome (NRY) of an exemplar sample. Long-read WGS data are shown in green. Short-read WGS and WES data are colored blue and red, respectively. In the ideogram of the Y chromosome, the heterochromatic regions (positive C-band) and the centromere are colored in gray and red, respectively. In the lowest panel, the pseudoautosomal regions (PAR1 and PAR2) and the NRY are represented in black and gray, respectively. To harmonize the results obtained from the three approaches, the depth of coverage was normalized to 100X. The R package karyotypeR v1.2.2 [38] was used to generate the depth of coverage plot.

The mean ( ± SD) number of NRY reads recovered per sample (n = 50) for short-read WGS and WES data were 8,329,867 ± 2,460,724 and 575,090 ± 176,201, respectively (Table S1). For WGS, 33.66% of the NRY showed at least 10X coverage. For WES, this percentage decreased to 0.90%. However, if only the exonic regions of the NRY were taken into account, 84.46% showed WES coverage of at least 10X. The mean ( ± SD) depth of coverage recovered across the NRY region for WGS was 13X ± 4 (range: 6–28X). The depth decreased to less than 1X for WES, although it was as high as 67X ± 19 (range: 27–111X) when only the exonic regions were included in the analysis. For the detected single nucleotide variants (SNVs), the mean ( ± SD) depth of coverage per SNV call was 60X ± 17 for WGS, decreasing to 32X ± 15 for WES. For ONT, the mean ( ± SD) number of NRY reads per sample (n = 8) recovered was 168,373 ± 36,333. The mean NRY depth of coverage was 12X ± 3 (range: 8–18X), and 33% of the NRY showed at least 10X coverage (Table S1). Furthermore, while the WGS data from both sequencing technologies provided a homogeneous depth of coverage profile across the NRY region (except in regions adjacent to the centromere and the heterochromatic region because of their complexity), the WES data showed a heterogeneous profile with enriched sites (peaks) associated with the capture of exons embedded within undetected regions (Fig. 1).

### 3.2 Consensus haplogroup classification

Based on the metrics retrieved for the short-read data, WGS showed higher values than WES for both the breadth and depth of coverage parameters, both of which are closely related to higher statistical support for variant detection. Therefore, we established the WGS-derived haplogroup as the ground truth. To assign the haplogroup of each sample, the haplogroup most frequently classified by all the assessed tools was used as the consensus haplogroup. Fourteen samples showed 100% concordance among the tools evaluated, and for the remaining samples, we obtained a mean concordance rate of 66.2% (Table S2). However, in most cases where discordance was observed, it was due to differences in haplogroup level classification and not to misclassification. In four of the samples, inconsistencies among the tools precluded a straightforward indication of the consensus haplogroup. In these four cases, the classification result of Yleaf was used as the ground truth given its higher classification accuracy demonstrated in all other samples. Considering only the WGS results, Yleaf offered the highest classification accuracy (94%). We found slightly lower but relatively high performance (>70%) for clean_tree_v2, Y-LineageTracker (with VCF as the input file type), HaploGrouper and pathPhynder. The worst-performing tool was Y-LineageTracker using BAM as the input file type since it misclassified 56% of the samples. Based on the limitations outlined in the methodology and in order to harmonize the results, the consensus haplogroup per sample used for the benchmarking was subordinated to the maximum level of resolution retrieved by WES (Table S2).

### 3.3 Haplogroup classification

The classification accuracy provided by short-read WGS data reached an average of 91%, while for WES, it decreased to an average of 54.8% (Table S2). On average ( ± SD), there was less cases with discordance among the WGS classifications (0.52 ± 0.87) than among the WES classifications (2.26 ± 0.85). On the basis of the classification accuracy for WGS data, Y-LineageTracker (VCF as the input file type) and Yleaf showed the highest accuracy, classifying precisely 98% and 96% of the analyzed samples, respectively. The following tools showed slightly lower accuracy: pathPhynder (92%), HaploGrouper (90%), and clean_tree_v2 (90%). Y-LineageTracker, using BAM as the input file type, was the least accurate tool for WGS data, misclassifying 20% of the samples. For WES data, Yleaf showed the highest classification accuracy among all tools, classifying precisely 92% of the analyzed samples. Clean_tree_v2 and pathPhynder had a slightly lower accuracy than Yleaf, with an average of 84%. HaploGrouper and Y-LineageTracker (VCF as the input file type) were the least accurate tools, yielding incorrect haplogroup classification in more than 88% of the samples. Y-LineageTracker (BAM as the input file type) did not provide any results for any of the samples because the tool could not identify any of the samples as male. This result is possibly related to the fragmented and noncontiguous nature of the exome data.

To assess the classification accuracy of ONT, the consensus haplogroup retrieved from short-read WGS data was used as the consensus haplogroup. The classification accuracy provided by long-read WGS reached an average of 83%, with a mean ( ± SD) of 1 ( ± 2) cases of discordance (Table S3). Among all the tools assessed, Yleaf, Y-LineageTracker (using BAM and VCF as the input file types), and pathPhynder offered the best performance, classifying precisely 88% of the analyzed samples. HaploGrouper and clean_tree_v2 showed slightly lower classification accuracy (75%).

### 3.4 Validation of the benchmarking results using alternative datasets

The mean ( ± SD) number of NRY reads recovered per sample (n = 54) for the 1KGP WGS and WES data were 6,835,617 ± 588,011 and 390,965 ± 270,653, respectively (Table S4). For WGS, 38.1% of the NRY showed at least 10X coverage, while for WES, this percentage was decreased to 0.6%. However, considering only the exonic regions of NRY, 68.2% showed at least 10X coverage by WES (Table S4). The mean ( ± SD) depth of coverage recovered across the NRY region for WGS was 14X ± 1 (range: 12–18X). The depth decreased to less than 2X for WES, although it was as high as 70X ± 35 (range: 29–200X) when only the exonic regions were included in the analysis. For the detected SNVs, the mean ( ± SD) depth of coverage per SNV call had a value of 77 ± 4 for WGS, decreasing to 12 ± 4 for WES.

Due to the limited classification resolution obtained by others for this dataset [39] based on the ISOGG nomenclature v9.06 (2016), we established the consensus haplogroup per sample based on ISOGG v15.73 (2020) using the same approach as described above. The classification accuracy provided by short-read WGS data reached an average of 88.6%, while for WES, it decreased to an average of 56.7% (Table S5). On average ( ± SD), there were more cases of discordance among the WES classifications (2.06 ± 0.79) than among the WGS classifications (0.68 ± 0.88). Regarding classification accuracy, Y-LineageTracker (VCF as the input file type) and Yleaf classified precisely 98.1% and 96.3% of the analyzed samples, respectively, based on WGS data. For WES data, pathPhynder, Yleaf, and clean_tree_v2 showed the highest classification accuracies among all tools, classifying precisely 90.7%, 88.9%, and 81.5% of the analyzed samples, respectively. Y-LineageTracker (with BAM as the input file type) showed the lowest accuracy for WGS data, classifying only 68.5% of the analyzed samples. HaploGrouper and Y-LineageTracker (VCF as the input file type) were the least accurate tools for WES data, yielding incorrect haplogroup classification in 88.9% of the analyzed samples.

### 3.5 Qualitative benchmarking of the haplogroup classification tools

Due to the numerous haplogroup classification tools available, an additional aim of this study was to guide researchers in selecting the most appropriate haplogroup classification tool for their analyses. To enable easy comparison among tools, a table outlining the advantages and limitations of each tool is provided for qualitative assessment. The following features were considered: haplogroup classification accuracy (taking the average of the classification results for the empirical and validation datasets), the ability to update the database used to the most recent version, the ability to process cohorts, versatility in the input files supported, the possibility of customizing parameter configuration, frequency of tool maintenance, and the inclusion of other major functions. To facilitate a comprehensive comparison among the different tools, the qualitative evaluation table (Table 2) highlights the advantages and limitations of each tool evaluated. Overall, Yleaf proved to be the most complete tool, demonstrating superior performance for more than 60% of the evaluated features. In contrast, HaploGrouper and clean_tree_v2 performed the worst of all the tools evaluated, with more than 50% of features classified poorly.

**Table 2:** Qualitative benchmarking of NRY haplogroup classification tools. The performance of each tool is evaluated across different features and represented on a color scale based on the level of performance: green triangle pointing upward for good, orange square for fair, and red triangle pointing downward for low performance. The haplogroup classification accuracy of each tool was categorized into three ranges based on the results for each application: tools with a haplogroup classification higher than 95% were considered to have good performance, fair performance was defined as a range between 90.00% and 94.99%, and low-performance tools were established as those with a classification rate lower than 89.99%. Regarding the database used, tools that allow the database to be updated to the latest version were represented as having fair performance, and tools that also allow more than one database (i.e., Yfull; https://www.yfull.com/tree) to be used were represented as having good performance. Multisample function was evaluated based on the possibility of cohort analysis. Tools that allow processing several samples as integrated functions or using a loop through a command line indicated good performance, and tools without this ability were represented as low performance. Based on the file formats supported, two categories were established: tools that support various input file formats were categorized as having good performance, and those that support only one file format were considered of low performance. The feature of allowing custom parameter configuration was divided into two categories: tools that did not allow parameterization were defined as having low performance, and tools that allowed optimization of certain parameters to improve classification accuracy were defined as having good performance. Tool maintenance was classified into two classes: tools updated continually or those that have been recently released were considered to have good performance, and tools that have not been updated in recent years were defined as having low performance. The last feature is the presence or absence of additional functions; tools that have other functions implemented are categorized as having good performance. The tools without more functions were determined to have low performance.

## 4 Conclusions

The advent of HTS technologies, the notable increase in the number of NRY polymorphic markers detected, and the importance of recovering ancestral information and pedigree relationships of study samples have motivated the development of new automated classification tools to adapt to these challenges. In this study, we present a benchmarking of five NRY haplogroup classification tools that could be easily upgraded to new versions of the ISOGG-Y-DNA tree. The comparison was carried out with empirical paired HTS data from WGS and WES, two of the most widely used applications in human genetics and medicine. In addition, paired WGS and WES data from 1KGP samples were used to validate the benchmarking results. The classification accuracy provided by each tool on the two datasets was consistent in most cases, demonstrating the validity of the results of this benchmarking study. Our results indicate that WES provides sufficient information to classify NRY haplogroups. However, tools employing VCF input files show a noticeable decrease in classification accuracy, which can be attributed to the low depth of specific sites that are excluded during the variant calling step. Based on this, to improve the classification accuracy for WES data or low-coverage WGS data, we recommend using a BAM file as the input file, given that this format contains all mapped reads. We demonstrate that Yleaf shows the best performance among all the tools evaluated for both applications, although with a slight loss in classification accuracy for WES data. In most of the samples, the classification retrieved matched that inferred by WGS, although in several samples, a lower level of accuracy was observed. However, considering that WES-derived sequences include a limited fraction of the NRY region, the performance achieved by the Yleaf tool for WES data is remarkable. Furthermore, Yleaf allows for custom configuration that can enhance the performance of the classification process in scenarios where the depth of coverage of the samples differs from the range assessed in our study. Regarding third-generation HTS, our findings show that despite the lower *per-base* accuracy currently offered by the assessed technology, it did not preclude equally accurate classification compared with that obtained from short-read data.

## CRediT authorship contribution statement

**Víctor García-Olivares:** Conceptualization, Data acquisition, Software, Formal analysis, Writing – Original draft preparation, Writing – Reviewing and Editing; **Adrián Muñoz-Barrera:** Conceptualization, Data acquisition, Software, Formal analysis, Writing – Original draft preparation, Writing – Reviewing and Editing; **Luis A. Rubio-Rodríguez:** Data acquisition, Formal analysis, Software, Writing – Reviewing and Editing**; David Jáspez:** Data acquisition, Formal analysis, Writing – Reviewing and Editing; **Ana Díaz-de Usera:** Data acquisition, Formal analysis, Writing – Reviewing and Editing; **Antonio Iñigo Campos:** Data acquisition, Formal analysis**; Krishna R. Veerama:** Data acquisition; **Santos Alonso:** Data acquisition, Writing – Reviewing and Editing**; Mark G. Thomas:** Data acquisition**; José M. Lorenzo-Salazar:** Data acquisition, Writing – Reviewing and Editing; **Rafaela González-Montelongo:** Data acquisition, Writing – Reviewing and Editing**; Carlos Flores:** Conceptualization, Data acquisition, Formal analysis, Writing – Original draft preparation, Funding acquisition, Writing – Reviewing and Editing.

## Conflicts of interest

The authors declare no conflict of interest.
