# Supplementary material for: Benchmarking of human Y-chromosomal haplogroup classifiers with whole-genome and whole-exome sequence data
Source: Comput Struct Biotechnol J. 2023 Sep 15;21:4613–8. doi: 10.1016/j.csbj.2023.09.012 (PMC10560978; doi:10.1016/j.csbj.2023.09.012)

Figure S1. Bioinformatic pipeline for data preprocessing and variant-calling of short-read WES/WGS and long-read WGS data of human NRY.

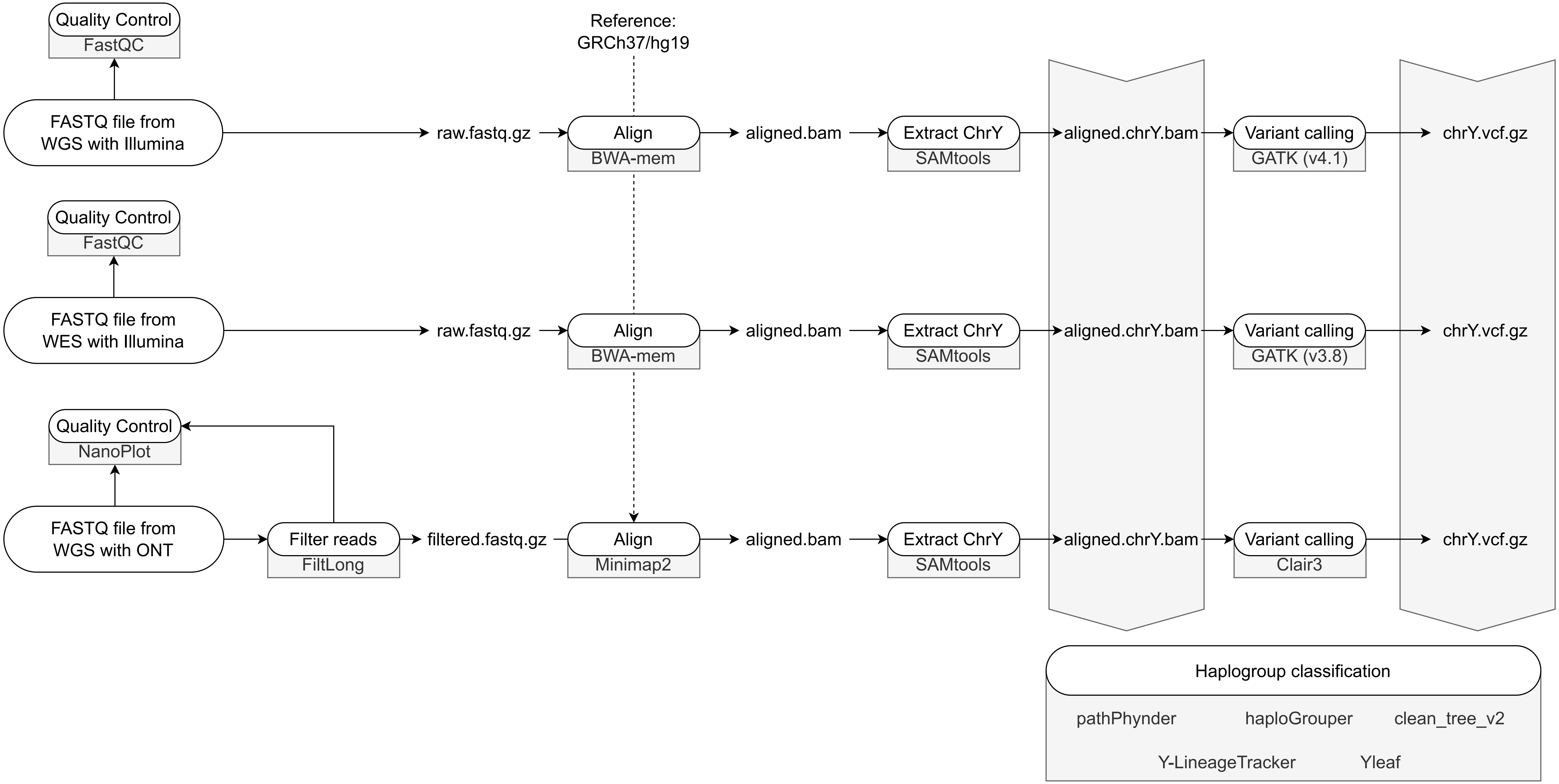

Supplement: Supplementary file 1 — Supplementary material [file mmc1.pdf]
